# Quantitative chest CT imaging characteristics and outcome of patients with COVID-19 associated pulmonary artery thrombosis: A single-center retrospective cohort study

**DOI:** 10.1097/MD.0000000000034250

**Published:** 2023-07-07

**Authors:** Cristian-Mihail Niculae, Adriana Hristea, Andreea Simona Albulescu, Vladimir Bogdan Petre, Ana-Maria-Jennifer Anghel, Anca-Cristina Damalan, Adela-Abigaela Bel, Mihai Lazar

**Affiliations:** a Faculty of Medicine, University of Medicine and Pharmacy “Carol Davila”, Bucharest, Romania; b National Institute for Infectious Diseases “Prof. Dr. Matei Bals”, Bucharest, Romania.

**Keywords:** COVID-19, pulmonary immunothrombosis, quantitative chest CT

## Abstract

Coronavirus disease 2019 (COVID-19)-associated pulmonary thrombotic events occur frequently and are associated with disease severity and worse clinical outcomes. We aimed to describe the clinical and quantitative chest computed tomography (CT) imaging characteristics based on density ranges (Hounsfield units) and the outcomes of patients with COVID-19 associated pulmonary artery thrombosis. This retrospective cohort study included all patients with COVID-19 hospitalized in a tertiary care hospital between March 2020 and June 2022 who underwent a CT pulmonary angiography. We included 73 patients: 36 (49.3%) with and 37 (50.7%) without pulmonary artery thrombosis. The in-hospital all-cause mortality was 22.2 versus 18.9% (*P* = .7), and the intensive care unit admission rates were 30.5 versus 8.1% (*P* = .01) at the time of diagnosis of pulmonary artery thrombosis. Except for D-dimers (median of 3142 vs 533, *P* = .002), the other clinical, coagulopathy, and inflammatory markers were similar. Logistic regression analysis revealed that only D-dimers were associated with pulmonary artery thrombosis (*P* = .012). ROC curve analysis of D-dimers showed that a value greater than 1716 ng/mL predicted pulmonary artery thrombosis with an area under the curve of 0.779, 72.2% sensitivity, and 73% specificity (95% CI 0.672–0.885). Peripheral distribution of pulmonary artery thrombosis was recorded in 94.5% of cases. In the lower lobes of the lungs, the incidence of pulmonary artery thrombosis was 6 times higher than that in the upper lobes (58–64%), with a percentage of lung injury of 80% to 90%. Analysis of the distribution of arterial branches with filling defects revealed that 91.6% occurred in lung areas with inflammatory lesions. Quantitative chest CT imaging provides valuable information regarding the extent of COVID-19 associated lung damage and can be used to anticipate the co-location of pulmonary immunothrombotic events. In patients with severe COVID-19, in-hospital all-cause mortality was similar regardless of the presence of associated distal pulmonary thrombosis.

## 1. Introduction

Severe acute respiratory syndrome coronavirus-2 (SARS-CoV-2) rapidly disseminated across the world since first cases were reported in Wuhan, China, in late December 2019, resulting in more than 6 million deaths worldwide.^[1]^ Coronavirus disease 2019 (COVID-19), the infectious diseases caused by SARS-CoV-2, is a complex respiratory and systemic disease, “orchestrated” by a severe and dysregulated proinflammatory response.^[2]^ As a result of the crosstalk between the immune and coagulation systems, viral-associated micro- and macro-vascular thrombotic events frequently occur especially in severe COVID-19 cases, being associated with worse clinical outcomes.^[2–4]^ Venous thromboembolism and COVID-19-associated coagulopathy, which includes patients without a positive imaging finding for thrombosis and abnormal coagulation parameters, especially D-dimers, are significant predictors of mortality among patients with severe SARS-CoV-2 infection.^[5–7]^ Additionally, older age, comorbidities, and a series of abnormal biomarkers of hematologic parameters, inflammation and cardiovascular injury are also associated with fatal outcomes in COVID-19 patients.^[7]^ The most common vascular thrombotic complication involves the pulmonary arteries, as a thrombosis that can be found in areas with inflammatory lung lesions (in situ immunothrombosis) and/or a microthrombotic pattern, as a diffuse occlusive thrombotic microangiopathy with alveolar damage.^[3,8]^ As segmental and/or subsegmental thrombi are more prevalent, evaluating the peripheral pulmonary arteries by a computed tomography (CT) pulmonary angiogram is essential in order to optimize the overall management of COVID-19 patients.^[9–16]^ Moreover, chest imaging data of patients with SARS-CoV-2 infection could be used to analyze the association between lung inflammatory lesions and in situ vascular thrombosis.^[8,14,17–19]^

In this study we aimed to describe clinical, quantitative chest CT imaging characteristics based on density ranges (Hounsfield units) and outcome of patients with COVID-19 associated pulmonary artery thrombosis. We used this term to cover pulmonary thromboembolism and also in situ immunothrombosis, as both mechanisms can contribute to COVID-19-associated macro-vascular thrombotic events.^[2,20]^

## 2. Material and methods

**Figure 1.**
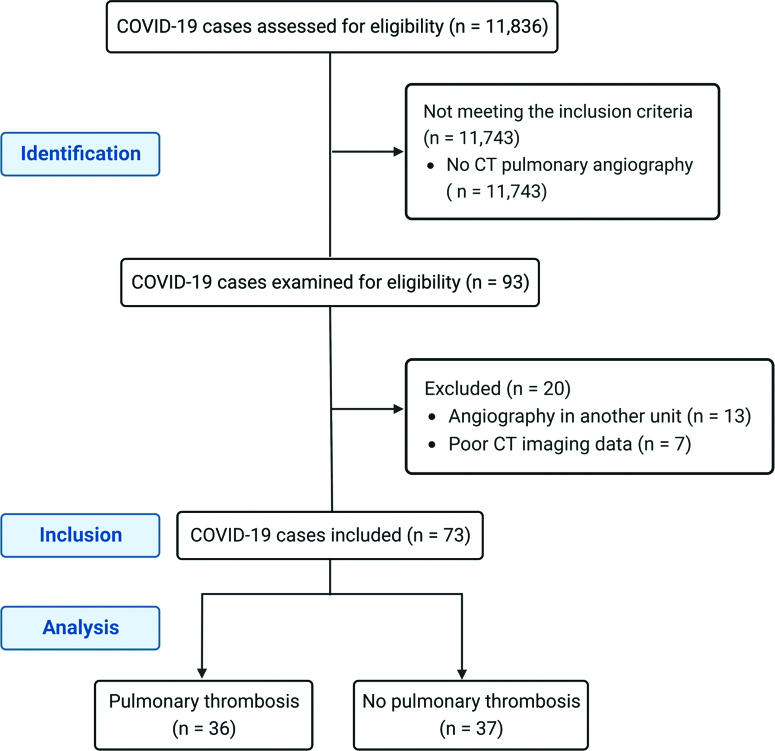
The flow diagram of the study describing the methods: recruitment of patients, inclusions and exclusions (created with Biorender.com).

We performed a retrospective observational cohort study that included hospitalized patients with COVID-19 between March 2020 and June 2022. We included all hospitalized adult patients over 18 years old with confirmed COVID-19 by Real-Time-Polymerase Chain Reaction and/or specific rapid antigen testing, who underwent a CT pulmonary angiography at clinical suspicion for the diagnosis of pulmonary artery thrombosis. We included only patients with the CT scan performed in our Radiology Department. We excluded patients with poor contrast enhancement of the pulmonary arteries and images with severe motion/breath artifacts. The flow diagram of the study is shown in Figure 1.

We defined COVID-19 disease severity as mild (normal oxygen saturation and no pulmonary lesions), medium (pneumonia on chest CT), or severe (based on at least one of the following additional criteria: peripheral oxygen saturation ≤ 93% in ambient air, respiratory rate > 30/minute, arterial oxygenation partial pressure to fractional inspired oxygen ratio < 300, or lung infiltrates > 50% of lung parenchyma).^[21]^ We assumed the PADUA Prediction Score for the identification of patients at risk for developing venous thromboembolic disease starting from 1 point, given COVID-19 infection. Reduced mobility was considered for patients who received supplemental oxygen therapy for at least 3 days. The percentage of lung involvement was calculated based on density ranges using a dedicated medical software (Syngo Pulmo3D, Siemens Healthcare GmbH, Erlangen, Germany, Volume). We considered alveolar lesions for densities > 0 Hounsfield units (HU), mixed lesions (alveolar and interstitial) for densities between 0 and −200 HU, interstitial lesions for densities between −200 and −800 HU, and normal lung densities between −800 and −1000HU.^[21]^ The CT scans were performed on a 64 slice CT Somatom Definition As (Siemens), with a rotation time 0.5 seconds, using a pitch of 1.2, collimation of 1.2 mm. CareDose4D and CareKV were used to reduce the radiation dose. The examination was performed with the patient in the head-first supine position in inspiratory breath hold. We injected a volume of 1 mL Omnipaque (300 mg I/mL)/kg in all patients included in the study (but not less than 70 mL), followed by a 50 mL saline bolus, using a flow of 3 mL/s. The scan start for the arterial phase was auto-triggered (bolus tracking) when the contrast in the pulmonary artery increased to 100 HU relative to that in the non-enhanced scan. The venous phase was performed with a delay of 20 seconds after the arterial phase. Primary diagnosis was performed on images with 5 and 3 mm thickness; and for multiplanar reconstructions we used 1.5 mm reconstructions with 0.5 mm overlap and B31f image filter, maximum intensity projection, and volume rendering images. To measure the density near the arterial branch with thrombi, a standardized ROI of 20 mm^2^ was used. Imaging review was blinded. The radiologist was blinded to the clinical and biological parameters registered for the patient.

Eligible patients were divided into 2 groups: group A (patients with pulmonary artery thrombosis) and group B (patients without pulmonary artery thrombosis). Clinical, laboratory, and imaging data of the enrolled patients were recorded when a diagnosis of pulmonary artery thrombosis was suspected by the clinician and were extracted from the medical and laboratory electronic charts. Descriptive data were expressed as frequencies (%) for categorical data, means ± Standard Deviation (SD) for continuous variables with normal distribution, and median and Interquartile Range (IQR) for continuous variables without normal distribution. Normal distribution was checked using a histogram and the Shapiro–Wilk test. Continuous variables were compared using the Mann–Whitney *U* test for data that significantly deviated from a normal distribution. A paired-sample *t* test was used for normally distributed continuous variables. Categorical variables were compared among groups using the chi-square test (or Fisher’s exact test if needed). Variables with >10% missing data were excluded from the analysis. Statistical significance was set at *P* < .05. Data were analyzed using the Statistical Package for Social Sciences (SPSS version 29, IBM Corp., Armonk, NY).

## 3. Results

### 3.1. Characteristics and outcome of patients with pulmonary artery thrombosis

We included 73 patients and divided them into 2 groups: group A (36 patients with pulmonary artery thrombosis) and group B (37 patients without pulmonary artery thrombosis). All the included patients had severe COVID-19. The clinical, biological, and imaging characteristics of both the groups are shown in Table 1.

**Table 1:** Clinical, biological and imaging characteristics of patients with and without pulmonary artery thrombosis.

Variables	Patients with pulmonary thrombosis N = 36	Patients without pulmonary thrombosis N = 37	*P* value
Clinical and biological characteristics			
Male, N (%)	27 (75)	26 (70.2)	.6
Age (yr)	62 (54–74)	66 (62.5–71.5)	.5
Comorbidities, N (%)	31 (86.1)	33 (89.1)	.7
Symptoms onset until hospital admission (d)	8 (7–10)	9 (7–11.5)	.6
Symptoms onset until CTPA (d)	15.5 (11–22)	15 (11.5–20)	.8
PADUA Prediction Score	4.6 ± 1.7	5.5 ± 1.4	.01
Wells score at CTPA	2.5 ± 2	2.3 ± 1.5	.6
Anticoagulated patients before CTPA, N (%)	28 (77.7)	30 (81)	.7
Duration of anticoagulation before CTPA (d)	5.5 (3–11.7)	6 (3.5–10)	.6
Leukocyte count (×10³/μL)	11.1 (8.5–16.8)	10.6 (7.3–13.8)	.8
Lymphocyte count (×10³/μL)	0.9 (0.4–1.3)	0.8 (0.5–1.2)	.8
Neutrophils/lymphocytes ratio	12.1 (5.7–23.2)	11.7 (6.2–18.5)	.8
Platelet count (×10³/μL)	236 (171–367)	266 (179–351)	.9
CRP (mg/L)	29 (6.8–75.6)	24.5 (4.2–67.7)	.9
Ferritin (ng/mL)	1029 (550–1650)	1068 (736–1558)	.4
IL-6 (pg/mL)	133.5 (42–505)	356 (84.7–1260.5)	.2
Fibrinogen (mg/dL)	362 (268–470)	346 (293–516)	.4
D-dimers (ng/mL)	3142 (1076–10459)	533 (297.5–2360)	.002
PT (s)	15.3 ± 9.5	15.3 ± 10.7	.9
PC (%)	79.5 ± 21.8	81.2 ± 20.7	.7
aPTT (s)	30.9 ± 9.3	34.3 ± 17.6	.3
LDH (U/L)	540 (371–712)	434 (390–569)	.2
ALT (U/L)	61 (34–109)	50 (35–92)	.4
AST (U/L)	53 (39–85)	44 (30–62)	.8
Need for ICU admission, N (%)	11 (30.5)	3 (8.1)	.01
Duration of the hospital stay (d)	22.5 (14–28)	19 (13–26.5)	.9
In hospital all-cause mortality, N (%)	8 (22.2)	7 (18.9)	.7
Quantitative chest CT imaging characteristics			
Pulmonary lobes with interstitial involvement	5 (5–5)	5 (5–5)	.4
Pulmonary lobes with alveolar involvement	3 (2–4)	2 (2–4)	.4
Pulmonary segments with interstitial involvement	19 (19–19)	19 (19–19)	.9
Pulmonary segments with alveolar involvement	5 (3–7)	4 (3–6)	.5
Interstitial pulmonary lesions (%)	52 (43.8–64)	49.6 (39.6–56.9)	.3
Alveolar pulmonary lesions (%)	9.7 (6.8–15.6)	9.8 (7–13.5)	.5
Total pulmonary lesions (%)	67.6 (56.8–79.4)	64.6 (53.2–76.8)	.5
Normal pulmonary densities (%)	32.4 (20.6–43.1)	35.4 (23.2–46.8)	.5
Pleural effusion, N (%)	9 (25)	10 (27)	.8
Pericardial effusion, N (%)	7 (19.4)	3 (11.1)	.1

Quantitative variables expressed as mean ± SD or median (IQR).

Laboratory test reference ranges: leukocytes 3.6–9.6 × 10^3^/μL, lymphocytes 1.2–3.4 × 10^3^/μL, platelet count 200–400 × 10^3^/μL, CRP < 3.0 mg/L, ferritin < 290 ng/mL, interleukin-6 < 7.0 pg/mL, fibrinogen 200–400 mg/dL, D-dimers < 230 ng/mL, PT 10.5–13.0 s, PC 80–115 %, aPTT 24–38 s, LDH < 246 U/L, ALT < 35 U/L, AST < 36 U/L.

ALT = alanine aminotransferase, aPTT = activated partial thromboplastin time, AST = aspartate aminotransferase, CRP = C-reactive protein, CTPA = CT pulmonary angiography, ICU = intensive care unit, IL-6 = interleukin-6, IQR = interquartile range., LDH = lactate dehydrogenase, PC = prothrombin concentration, PT = prothrombin time, SD = standard deviation.

Although the 2 groups were similar in terms of age, sex, comorbidities, clinical findings, Wells score, and anticoagulant treatment, patients in group B were at a higher risk of developing thrombosis according to the PADUA prediction score (4.6 vs 5.5, *P* = .01). Only one patient in the pulmonary artery thrombosis group had clinical signs of deep venous thrombosis. Except for D-dimers (3142 vs 533, *P* = .002), coagulopathy and inflammatory markers (prothrombin time, prothrombin concentration, activated partial thromboplastin clotting time, fibrinogen, platelets, leukocyte cell count, C-reactive protein [CRP]) were not significantly different between the 2 groups. Receiver operating characteristic curve (ROC curve) analysis of D-dimers showed that a value greater than 1716 ng/mL predicted pulmonary artery thrombosis with an area under the curve of 0.779, 72.2% sensitivity, and 73% specificity (95% CI 0.672–0.885). The number of pulmonary lobes/segments with interstitial and alveolar involvement and the percentage of interstitial/alveolar pulmonary lesions were also similar between the 2 groups. Logistic regression analysis revealed that higher D-dimer levels were associated with pulmonary artery thrombosis (*P* = .012). The other variables assessed were sex (*P* = .3), age (*P* = .9), presence of comorbidities (*P* = .6), CRP (*P* = .7), ferritin (*P* = .3), IL-6 (*P* = .3), and the percentage of total pulmonary lesions (*P* = .5).

Pulmonary artery thrombosis was associated with the need for mechanical ventilation and intensive care unit admission [11 (30.5%) vs 3 (8.1%), *P* = .01], but not with higher all-cause mortality rates [8 (22.2) vs 7 (18.9), *P* = .7] during hospitalization.

### 3.2. Imaging characteristics of patients with pulmonary artery thrombosis

**Figure 2.**
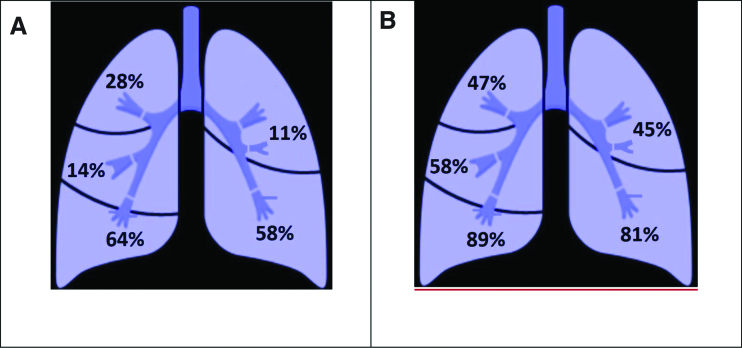
Distribution of pulmonary artery thrombosis in the study group (A); percentage of inflammatory lesions (B).

All patients with pulmonary artery thrombosis presented filling defects in the segmental branches of the pulmonary artery, 13 (35%) patients in the lobar branches, 2 (5.5%) patients in the left/right pulmonary artery, and none of the patients presented with involvement of the main pulmonary artery. Twenty-one patients (58%) presented with thrombosis in the left inferior lobe, 4 (11%) patients in the left superior lobe, 23 (64%) patients in the right inferior lobe, 5 (14%) patients in the right middle lobe, and 10 (28%) patients in the right superior lobe (Fig. 2A). The distribution of total lung lesions showed more severe involvement of the lower lobes compared to the upper lobes (Fig. 2B), concordant with the higher incidence of pulmonary artery thrombosis in the left and right lower lobes.

Nineteen (52.8%) patients presented with involvement of 1 pulmonary lobe, 11 (30.5%) patients with involvement of 2 lobes, 3 (8.3%) patients with involvement of 3 lobes, 2 (5.5%) patients with involvement of 4 lobes, and 1 (2.7%) patient with thrombosis in all 5 pulmonary lobes. Pulmonary artery thrombosis was mostly described in the posterior segments of the right inferior lobe 20 (56%) patients, followed by the posterior segment of the left inferior lobe 19 (53%) patients and lateral segment of the right inferior lobe 16 (44%) patients; the distribution of pulmonary artery thrombosis on pulmonary segments is further detailed in Table 2.

**Table 2:** Distribution of pulmonary artery thrombosis on pulmonary segments.

Pulmonary lobe N (%)	Pulmonary segments	Patients with thrombosis N (%)
Left inferior lobe, 21 (58)	Superior	2 (6)
	Posterior	19 (53)
	Lateral	9 (25)
	Anterior	4 (11)
Left superior lobe, 4 (11)	Superior lingular	1 (3)
	Inferior lingular	1 (3)
	Superior	2 (6)
	Posterior	0
	Anterior	3 (8)
Right inferior lobe, 23 (64)	Superior	3 (8)
	Posterior	20 (56)
	Lateral	16 (44)
	Anterior	6 (17)
	Medial	4 (11)
Right middle lobe, 5 (14)	Medial	4 (11)
	Lateral	2 (6)
Right superior lobe, 10 (28)	Superior	9 (25)
	Anterior	4 (11)
	Posterior	4 (11)

The density measurement adjacent to the arterial branch with pulmonary artery thrombosis revealed densities suggestive of alveolar consolidation in 3 patients, densities suggestive of interstitial involvement in 30 patients, and normal densities in 3 patients, with an average density of −479 HU. Although the patients with thrombosis presented a median percentage of 32.4% of lung parenchyma without inflammatory lesions, in 91.6% of cases, it was detected in the lung parenchyma with interstitial or alveolar lesions.

## 4. Discussion

This retrospective study included 73 hospitalized patients with severe COVID-19 who underwent a CT pulmonary angiography because of clinical suspicion of pulmonary artery thrombosis. Consistent with the data from the literature, the incidence of pulmonary artery thrombosis was 49.3%. In a systematic review and meta-analysis, the incidence was 18% to 57%, with a pooled determined incidence of 30.2% [95% CI: 21.0–41.3].^[22]^ We found a significant difference in D-dimers values between patients with pulmonary artery (macro)thrombosis and the control group (3142 vs 533, *P* = .002). Similar to our study, in 2 other studies involving patients with SARS-CoV-2 associated pneumonia, a significant difference in median values of D-dimers was recorded between patients with and without pulmonary thrombosis (9841 vs 1285, *P* < .001) (3725 vs 1754 ng/mL, *P* < .01).^[18,23]^ Various cutoffs for D-dimer have been suggested with differing specificities and sensitivities for the diagnosis of pulmonary artery thrombosis. In our study, ROC curve analysis of D-dimer showed that a value greater than 1716 ng/mL predicted pulmonary artery thrombosis with 72.2% sensitivity and 73% specificity. Similarly, Cau et al, in an analysis of 84 patients, identified, based on ROC analysis of D-dimer, that a value greater than 1929 ng/mL predicted pulmonary artery thrombosis with an area under the curve of 0.728, 67% sensitivity, and 70% specificity (95% CI, 0.620–0.840).^[18]^ Other authors found that D-dimer levels above 5000 ng/mL had better specificity (89%) with a similar sensitivity of 73%, but the patients in that study were more severe, presenting with acute respiratory distress syndrome associated with COVID-19.^[24]^ While sensitivity is certainly significant for the rapid detection and treatment of pulmonary artery thrombosis events, a greatly reduced specificity may result in misdiagnosis and unnecessary use of hospital resources, especially in centers with limited medical funding. It has also been suggested that D-dimer levels could be used to discriminate between patients with a high risk of clinical deterioration and can establish an indication for anticoagulation, especially for patients with non-subsegmental thrombosis.^[25]^

We did not find a significant difference between the 2 groups of patients regarding systemic inflammatory markers (CRP, ferritin, IL-6 levels), as found in 2 large cohorts of 575 and 697 patients with COVID-19 and pulmonary thrombosis.^[26,27]^ Regarding age, sex, and comorbidities, our results were similar to those of the aforementioned studies, with no difference between the 2 groups.

More than one-third of the patients had at least 2 to 5 pulmonary lobes involved, as a diffuse pulmonary artery thrombosis. Local lung inflammation on quantitative chest CT imaging found an incidence of pulmonary artery thrombosis of 10% to 11% for a percentage of lung injury lower than 50% in the upper lung lobes. The incidence was 6 times higher (58–64%) for a percentage of lung injury of 80% to 90% in the lower lobes. Also, our analysis of the distribution of arterial branches with filling defects revealed that 91.6% occurred in alveolar lung areas with inflammatory lesions. Patients with SARS-CoV-2 infection have associated thrombosis located in the lung parenchyma with inflammatory lesions, including ground-glass opacities, consolidation, and “crazy paving.”^[8,17]^ In their study, Mueller-Peltzer et al^[8]^ found that all thrombi were located in segments with COVID-19-associated inflammatory lesions. Similarly, in another paper, 87% of patients had pulmonary thrombosis in the lung parenchyma affected by SARS-CoV-2 pneumonia.^[18]^ As in our study, chest CT analysis of patients with pulmonary artery thrombosis shows significantly higher percentage of SARS-CoV-2-associated inflammatory lesions especially in the lower lobes of the lungs, where blood clots predominate.^[8,14,19]^ These findings suggest a possible association between vascular thrombosis and inflammatory lung changes that could lead to in situ immunothrombosis. The low response to systemic anticoagulation for preventing pulmonary artery thrombosis in most of our patients (77.7% for a median of 5.5 days) could further support this theory, which has also been reported in other clinical and pathological studies of COVID-19.^[14,28,29]^

We found no difference between in-hospital all-cause mortality of patients with and without pulmonary artery thrombosis (22.2 vs 18.9, *P* = .7), similar to data from 2 large cohorts of COVID-19 patients.^[26,30]^ This might be due to the fact that both groups of patients had severe disease, associated coagulopathy and the pulmonary thrombosis affected distal peripheral arteries. Our data are in contrast with other studies, in which quantitative chest CT data regarding the extent of pulmonary thrombosis were missing and/or not all included patients had severe COVID-19.^[31,32]^ In another large cohort of COVID-19 patients, mortality rates were different between patients with and without pulmonary thrombosis (42.5 vs 32.3%, *P* = .009), but in most cases (64.2%), proximal, trunk, or main pulmonary involvement was recorded.^[27]^ Associated coagulopathy could also play an important role in disease evolution in severe COVID-19, even if pulmonary artery thrombosis is not detected. This is important in clinical practice, as in patients with extensive lung involvement, as there are many factors that can limit the image quality and depiction of filling defects in the pulmonary artery branches, especially the distal vessels.^[8]^ Therefore, the value of CT in the detection of pulmonary artery thrombosis is important, especially for large arterial branches; however, a negative result should not automatically exclude the diagnosis of distal thrombosis, especially in the context of positive clinical and biological markers. D-dimer cutoff values could be an objective method for clinical decision-making when imaging is not available.^[25]^ Moreover, according to current guidelines, therapeutic anticoagulation is recommended for hospitalized COVID-19 patients with supplemental oxygen needs outside the intensive care unit, even if not diagnosed with pulmonary artery thrombosis.^[33,34]^ Our findings support this recommendation.

Limitations of this study include its retrospective nature and small sample size because of the limited access to imaging investigations during the COVID-19 pandemic. There could also be subclinical pulmonary artery blood clots, as not all patients with COVID-19 were screened by CT pulmonary angiography. Additionally, we were unable to account for the potential impact of some underlying prothrombotic genetic risk factors in the patients with thrombosis.

## 5. Conclusions

Quantitative chest CT imaging provides valuable information regarding the extent of COVID-19 associated lung damage and can be used to anticipate the co-location of pulmonary immunothrombotic events. When patients with severe COVID-19 have associated peripheral blood clots, the in-hospital mortality rates are similar to those with severe disease and coagulopathy but without pulmonary artery thrombosis. In terms of clinical practice and optimal case management, a similar anticoagulant-type approach is necessary for these patients, as recommended by the current guidelines.

## Acknowledgments

This work is part of the PhD thesis “Clinical and imaging evolutionary aspects in moderate and severe forms of SARS-CoV-2 infection,” performed at the University of Medicine and Pharmacy “Carol Davila,” Bucharest, Romania. Publication of this paper was supported by the University of Medicine and Pharmacy “Carol Davila,” through the institutional program “Publish not Perish”;

## Author contributions

**Conceptualization:** Cristian-Mihail Niculae, Adriana Hristea, Mihai Lazar.

**Data curation:** Cristian-Mihail Niculae, Andreea Simona Albulescu, Vladimir Bogdan Petre, Ana-Maria-Jennifer Anghel, Anca-Cristina Damalan, Adela-Abigaela Bel, Mihai Lazar.

**Formal analysis:** Cristian-Mihail Niculae, Andreea Simona Albulescu, Vladimir Bogdan Petre, Mihai Lazar.

**Investigation:** Cristian-Mihail Niculae, Adriana Hristea, Andreea Simona Albulescu, Vladimir Bogdan Petre, Ana-Maria-Jennifer Anghel, Mihai Lazar.

**Methodology:** Cristian-Mihail Niculae, Adriana Hristea, Anca-Cristina Damalan, Adela-Abigaela Bel, Mihai Lazar.

**Project administration:** Adriana Hristea.

**Resources:** Cristian-Mihail Niculae, Andreea Simona Albulescu, Vladimir Bogdan Petre, Ana-Maria-Jennifer Anghel, Anca-Cristina Damalan, Adela-Abigaela Bel.

**Software:** Cristian-Mihail Niculae, Andreea Simona Albulescu, Vladimir Bogdan Petre, Mihai Lazar.

**Supervision:** Adriana Hristea, Mihai Lazar.

**Validation:** Adriana Hristea.

**Visualization:** Anca-Cristina Damalan, Mihai Lazar.

**Writing – original draft:** Cristian-Mihail Niculae, Andreea Simona Albulescu, Vladimir Bogdan Petre, Mihai Lazar.

**Writing – review & editing:** Cristian-Mihail Niculae, Adriana Hristea, Mihai Lazar.
